# Research on the Collision Risk of Fusion Operation of Manned Aircraft and Unmanned Aircraft at Zigong Airport

**DOI:** 10.3390/s24154842

**Published:** 2024-07-25

**Authors:** Longyang Huang, Chi Huang, Chao Zhou, Chuanjiang Xie, Zerong Zhao, Tao Huang

**Affiliations:** 1 College of Air Traffic Management, Civil Aviation Flight University of China, Deyang 618307, China; longyanghuang@cafuc.edu.cn; 2 Institute of Electronic and Electrical Engineering, Civil Aviation Flight University of China, Deyang 618307, China; zc_cafuc@163.com (C.Z.); 18428327587@163.com (C.X.); 15520664026@163.com (T.H.); 3 Mianyang Branch, Civil Aviation Flight University of China, Mianyang 621052, China; zzrhappiness@163.com

**Keywords:** unmanned aircraft, fusion operation, collision risk, safety spacing margin, Monte Carlo simulation

## Abstract

Low-altitude airspace is developing rapidly, but the utilization rate of airspace resources is low. Therefore, in order to solve the problem of the safe operation of the fusion of large UAVs and manned aircraft in the same airspace, this paper analyzes the theoretical calculation of the collision risk of the fusion operation of manned aircraft and UAVs at Feng Ming Airport in Zigong, verifying that while assessing the safety spacing of 10 km in the lateral direction, it further simulates the possibility of calculating the theoretical smaller safety spacing. The study will propose a new theory of error spacing safety margin and improve it according to the traditional Event collision risk model, combining the error spacing safety margin to establish an improved collision model more suitable for the fusion operation of manned and unmanned aircraft and reduce the redundancy of calculation. The error factors affecting manned and unmanned aircraft at Zigong Airport are analyzed, and theoretical calculations are analyzed by combining the actual data of Zigong Airport. Finally, the Monte Carlo simulation method is used to solve the error, substitute the calculation results, and simulate a section of the trajectory of the fusion operation for the reverse argument. The theoretical calculation results show that the collision risk from 10 km to 8 km satisfies the lateral target safety level (TSL) specified by ICAO under both traditional and improved models. The collision risk calculated by the improved model incorporating the error spacing safety margin is smaller, which enhances the safety of the model calculations. The results of the study can provide theoretical references for the fusion operation of manned and unmanned aircraft.

## 1. Introduction

Currently, the reform of low-altitude airspace management is gradually deepening, fostering growth in the comprehensive industry centered on the low-altitude economy [1]. Among the challenges faced are the rapid development of unmanned aircraft, the low utilization rate of low-altitude airspace resources, and their uneven distribution. Additionally, the scenario of sharing airspace between unmanned and manned aircraft is under active research and exploration [2].

In 2024, the State Council, in conjunction with the Central Military Commission, issued the Interim Regulations for the Administration of Unmanned Aerial Vehicle Flight, among other documents, highlighting the aviation industry as a key direction for inclusion in the development of the country’s strategic emerging industries. These documents emphasize the orderly opening of low-altitude airspace and the continuous promotion of constructing and operating unmanned aerial test areas [3]. Presently, China’s low-altitude airspace typically refers to airspace below 3000 m or below 1000 m in true height [4]. Within this low-altitude airspace, UAVs are applied in various sectors, including aerial photography [5], power inspection [6], and pesticide spraying [7], as well as in larger applications such as enemy detection and combat [8], forest fire-fighting, and communication rescue operations [9]. Meanwhile, the dominant manned aircraft in low-altitude airspace are often training models, such as those used by the Civil Aviation Flight Academy of China (CAFA). The continuous opening of low-altitude airspace, along with the development of electric vertical takeoff and landing (eVTOL) vehicles and other flying vehicles, has positioned urban air transportation as a sustainable and green direction for future transportation [10]. However, due to differences in airspace jurisdiction and policies across countries, domestic research on the integration of manned aircraft and UAVs is still in its infancy. There remain many challenges to be addressed to enable UAVs to operate in the same airspace as manned aircraft, necessitating robust theoretical research to support practical operations. Therefore, integrating various UAVs into the airspace of manned aircraft presents complex air traffic management problems, making collision risk assessment of integrated operations a critical research direction.

In 1964, Reich [11] proposed the well-known Reich model for long-range air traffic systems, based on aircraft position, speed, and random deviations. The model aims to analyze and determine safe air traffic separation criteria to ensure sufficient spacing between aircraft to absorb systematic speed differences as well as imperfections in navigation and piloting, known as flight errors [12,13]. Cui et al. [14,15] addressed the collision risk of large unmanned aerial vehicles (UAVs) operating in converged airspace. They improved the Reich model, considered the operational and error characteristics of large UAVs, and modeled the collision between UAVs and manned aircraft. By selecting the existing Beijing–Shanghai route for simulation, the collision risks before and after joining UAVs in the existing airspace are calculated and compared, and the feasibility of large UAVs entering the fused airspace is discussed.

With further research, Brooker [16] proposed a new collision risk model, the Event collision risk model, for longitudinally separated aircraft in the ATC trajectory system in the North Western Ocean region, based on aircraft position, speed, and maneuvering characteristics, which simplifies adjustments to the minimum inter-aircraft separation time and provides a safety assessment for improving airspace use efficiency. Lili Wang [17] and others proposed a new collision risk assessment model for the operational safety of small UAVs in low-altitude airspace, based on the randomly distributed velocity characteristics of UAVs. The model takes into account the maneuvering flexibility of UAVs and the rapidity of speed changes, and designs a double-layer sphere collision template for free-flying UAVs and a rectangular body collision template for UAVs flying along a fixed path, which more accurately evaluates the collision risk of UAVs in low-altitude airspace. In 2017, Junqiang Wan et al. [18] proposed a new longitudinal collision risk assessment model based on CNS performance and human factors for aircraft in parallel flight paths. By introducing the CREAM method and considering the controller’s intervention behavior and human cognitive reliability, the model provides a new perspective and analysis method for the study of collision risk between aircraft [19]. In 2019, Huang et al. [20] proposed a series of collision avoidance control strategies for the collision avoidance problem in multi-UAV systems, based on the position, velocity, and random deviation of UAVs. These strategies cover from the path planning of a single UAV to the cooperative collision avoidance control of a multi-UAV team, aiming to provide theoretical support and practical guidance for the safe operation of UAVs in complex environments. In 2021, Chen Yi-You [21] and Yu Qing-Yuan et al. [22] addressed the safe operation of UAVs in the manned shared airspace by proposing a relevant model and methodology, which provides risk assessment and spacing optimization for the integration of UAVs into the civil aviation airspace management system, providing a new method for risk assessment and spacing optimization. In 2022, Kallinen et al. [23] extended the classical Reich collision risk model for UAVs in non-separated airspace to assess and optimize the operational risk of UAVs in non-segregated airspace in response to the lateral separation criterion for UAV traffic management based on the UAV’s position, speed, and navigational performance. In 2023, Figuet [24] proposed a novel data-driven approach combining Monte Carlo simulation and the Peaks Over Threshold (POT) method [25] from Extreme Value Theory [26] (EVT) to estimate the probability of an aircraft airborne collision. Zhou et al. [27] proposed the Unmanned and Manned Aircraft-Event Modeling collision risk model for the lateral safety spacing problem in the mixed mode of UAV and manned aircraft operation. The improved model is based on the event collision risk model and comprehensively considers various factors such as communication, navigation, surveillance performance, human factors, performance of collision avoidance equipment, and meteorological conditions. This enhancement provides a theoretical basis and practical guidance for determining safe spacing in the integrated operation of unmanned and manned aircraft. These studies primarily focus on improving collision risk models, particularly for assessing the collision risk of large drones operating in integrated airspace. Additionally, they involve the development of new collision risk models considering factors such as aircraft speed, CNS performance, and human factors, as well as the study of safe operation strategies for multiple drone systems. These studies have filled the research gap in the optimization of safe separation distances for drone operations. However, there is still a lack of research in evaluating the safe separation distances for the integrated operation of manned and unmanned aircraft, as well as the impact of positioning errors.

Therefore, this paper aims to evaluate the feasibility of safe operations for drones and manned aircraft within the same airspace, addressing the practical needs of low-altitude airspace development. Utilizing the team’s previous research, we establish an integrated operation event collision risk model and an improved integrated operation model, representing aircraft with a combination of rectangular and cylindrical shapes. Factors such as navigation errors and meteorological condition errors are incorporated to innovatively propose error safety margin distances, establishing reasonable error protection zones for aircraft. Using the operation scenarios and data from the Zigong Airport experimental area, we study the collision risk of integrated operations between drones and manned aircraft, assessing the feasibility of a 10 km safe separation distance at Zigong Airport and exploring the theoretical possibility of reducing this distance to 8 km. Finally, we employ the Monte Carlo simulation method to solve the deviation of the error margin distance and perform trajectory simulation predictions for manned and unmanned aircraft under the influence of added errors. Through the analysis and calculation of collision risks, we understand and verify the flight safety under different safety distance conditions. These research findings are crucial for ensuring the safety of manned and unmanned aircraft in air traffic.

## 2. Integration of Operational Collision Risk Models

### 2.1. Event Crash Risk Model

**Figure 1:**
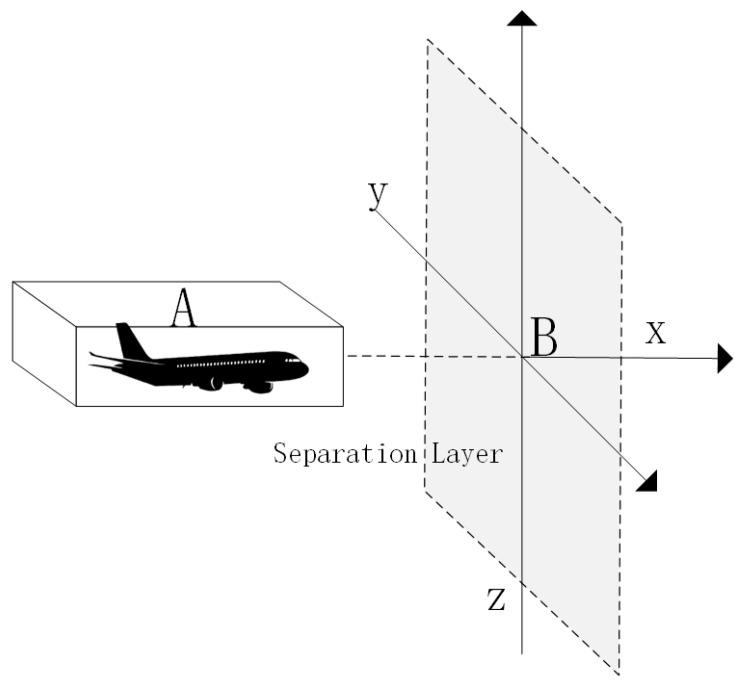
Event crash risk model.

Collision risk refers to the risk of collision that occurs when the minimum safe distance between aircraft cannot meet the regulations due to various types of errors during flight [28]. The Event collision risk model is a classic and widely used collision risk assessment model, which is a collision risk assessment methodology developed by the Federal Aviation Administration (FAA) of the United States. The model is mainly based on the parameters of distance, time, and speed of two aircraft to establish a motion model for calculation [29]. Not only that, other impact parameters can be added to the Event model, and the model can also be optimized and improved. Therefore, this paper chooses this model as the research base model to analyze the collision risk of fusion operation of manned aircraft and unmanned aircraft, and its model is schematically shown in Figure 1.

**Figure 2:**
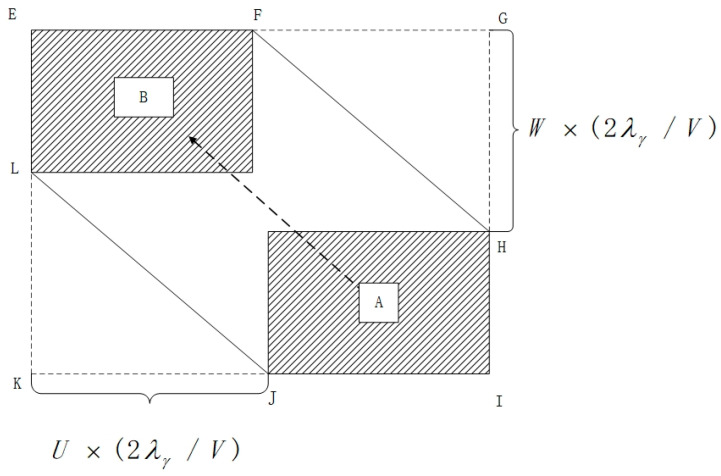
Crash box.

As shown in the model in Figure 1, a rectangular collision box is built for manned aircraft A, and UAV B is regarded as the collision surface, where the length, width, and height of the rectangle represent the collision risk in the lateral, longitudinal, and vertical directions. Therefore, the probability of lateral collision risk between the two aircraft is equal to the product of the probability that UAV B is located in the extended collision box and the probability that manned aircraft A passes through the interval laterally, as shown in Figure 2.

As shown in Figure 2, in calculating the collision risk, the motion models of the two aircraft need to be modeled first. Then, the two motion models are superimposed to obtain the relative motion model. Next, the expanded collision box is added to the relative motion model to estimate the collision risk more accurately. Finally, the collision risk assessment results are derived by calculating the number of target airplanes in the expanded collision box and the time interval. In Figure 2, A represents the projected area of the manned aircraft model collision box, B represents the projected area of the unmanned aircraft model collision box, HG represents the vertical distance, and KJ represents the longitudinal distance. The specific calculation formula is shown in Equation (1):

(1) $$N_{y}=2G_{y}E_{x}P_{z}\frac{\lambda_{x}}{S_{y}}(1+\frac{2v_{x}\lambda_{y}}{2v_{y}\lambda_{x}})(1+\frac{2v_{z}\lambda_{y}}{2v_{y}\lambda_{z}})$$

In Equation (1), $N_{y}$ represents the lateral collision risk probability; $G_{y}$ is the lateral interval loss rate; $E_{x}$ is the longitudinal proximity rate; $P_{z}$ is the vertical overlap probability; λ are the length, wingspan, and height of UAV A, respectively; $S_{y}$ is the area of the shaded portion of the collision box; and V are the relative velocities of the two aircrafts in the longitudinal, lateral, and vertical directions, respectively.

### 2.2. Improved Crash Risk Modeling

**Figure 3:**
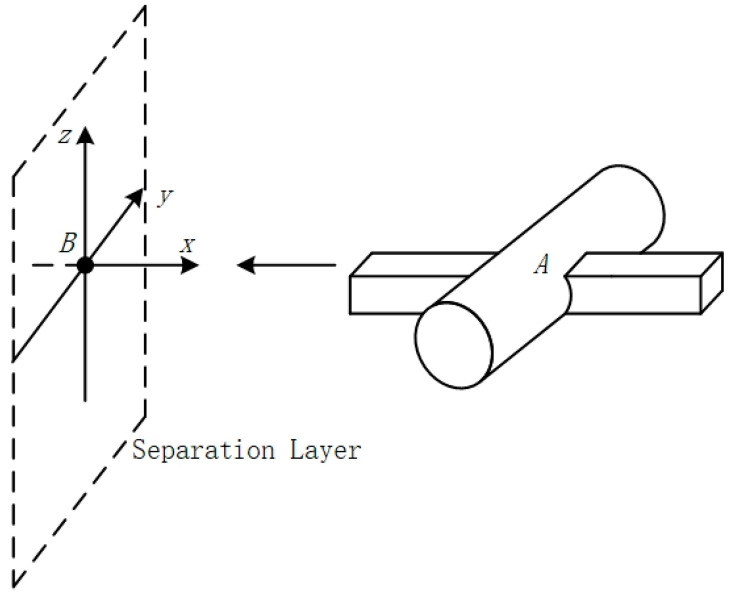
Improved crash risk modeling.

The Event model defines the collision box of an aircraft as a rectangular body, but analyzing the collision probability of an aircraft in flight through a rectangular body results in a large amount of computational redundancy causing computational bias [30]. In order to improve the calculation, an improved collision risk model that is more suitable for change compared to the traditional model is used [31]. In the improved geometry collision risk model, A is used as the geometric model of the UAV, where the area of the cylinder A is defined as the cross-sectional area of the collision surface of the UAV. The geometric center of the manned aircraft B is used as the origin to establish a spatial right-angled coordinate system, where the x, y, and z axes denote the longitudinal, lateral, and vertical spacers of the relative motion between the aircraft, respectively. The longitudinal spacing layer plane is the plane obtained by crossing the *y*-axis and *z*-axis. The specific schematic diagram is shown in Figure 3.

**Figure 4:**
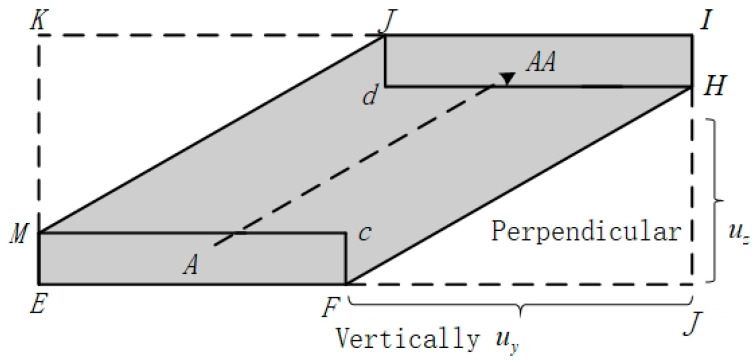
Model projection view.

As shown in Figure 3, the fuselage of aircraft A is represented by a combination of cylinders and rectangles, and the improved model is more suitable for inter-aircraft collision risk calculations, reduces computational redundancy, and improves the accuracy of the theoretical analysis, relative to the traditional Event collision risk model with a rectangular collision box [32]. This is due to the simple geometry of rectangular and cylindrical bodies, which are easy to model and compute collision detection while providing a reasonable approximation of the main structural features of the aircraft. The rectangular body is suitable for representing the elongated fuselage, while the cylinder is suitable for representing the wings and tail. This simplified model improves computational efficiency while maintaining sufficient accuracy, making collision risk assessment more efficient and practical. See Figure 4.

Assuming that the overall area of the shaded portion is $S_{y}$, the area of the rectangle EJIK is $S_{c}$, JH is the distance flown vertically by the UAV as it traverses the collision layer, and FJ is the distance traveled by the UAV in the longitudinal direction, thus:

(2) $$JH=u_{z}\times \frac{2\lambda_{y}}{u_{y}}$$

(3) $$FJ=u_{x}\times \frac{2\lambda_{y}}{u_{y}}$$

From this, it follows that:

(4) $$S_{c}=\left(2\lambda_{x}+u_{x}\times 2\frac{\lambda_{y}}{u_{y}}\right)\times \left(2\lambda_{z}+u_{z}\times 2\frac{\lambda_{y}}{u_{y}}\right)$$

Then, the area of the shaded portion is:

(5) $$S_{y}=(2\lambda_{x}+u_{x}\times \frac{2\lambda_{y}}{u_{y}})\times \left(2\lambda_{z}+u_{z}\times \frac{2\lambda_{y}}{u_{y}}\right)-u_{z}\times \frac{2\lambda_{y}}{u_{y}}\times u_{x}\times \frac{2\lambda_{y}}{u_{y}}$$

According to the theory of traditional Event collision modeling, the lateral collision risk $N_{y}$ between UAV and manned aircraft flying at the same altitude can be obtained as Equation (6):

(6) $$N_{y}=2G_{y}E_{x}P_{z}\times \frac{\lambda_{z}}{S_{z}}\times \frac{u_{y}}{2\lambda_{y}}\times (\frac{2u_{z}\lambda_{y}\times 2u_{y}\lambda_{z}}{2u_{y}\lambda_{z}})\times (\frac{2u_{y}\lambda x\times 2u_{x}\lambda_{y}}{2u_{y}\lambda_{x}})\times \frac{S_{y}}{S_{c}}$$

where $N_{y}$ represents the probability of lateral collision risk; $G_{y}$ is the loss rate of lateral spacing; $E_{x}$ is the longitudinal proximity rate; $P_{z}$ is the probability of vertical overlap; λ is the representative airplane length, wingspan, and height, respectively; $S_{y}$ is the area of the shaded part of the collision box; $S_{c}$ is the area of the collision box; and $u_{x},u_{y},u_{z}$ are the relative speeds of the two airplanes in the longitudinal, lateral, and vertical directions, respectively.

### 2.3. Error Safety Distance Margin

**Figure 5:**
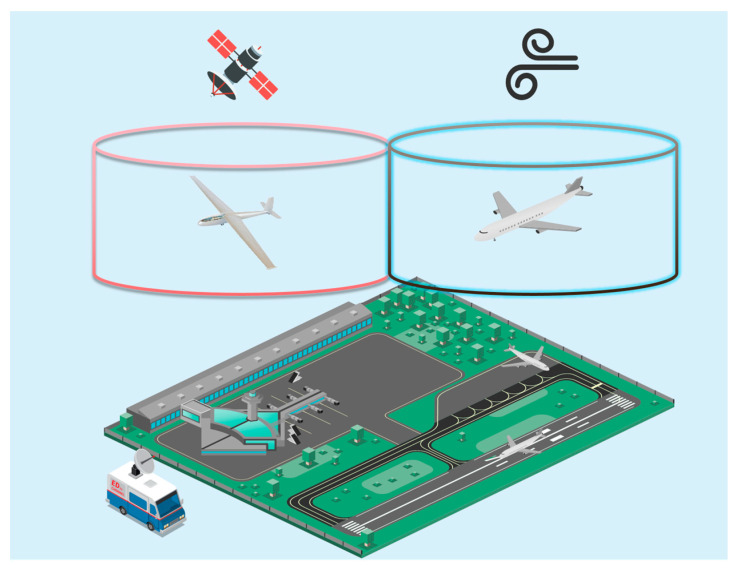
Schematic diagram of the safety margin model.

There are multiple error factors in the fusion operation of manned and unmanned aircraft, and these errors are random and uncertain [33]. In order to ensure the safe and effective operation of the aircraft, the study in this paper innovatively proposes to set up an error spacing safety margin around the aircraft model, which is integrated into the final calculation of the model. Considering that the UAVs in fusion operation are mainly large UAVs, which usually fly with fixed route, and assuming that ε denotes the total error and σ denotes various localization error factors, the projected cross-sectional area of the UAV can be mainly regarded as a circle with a radius of R. Therefore, when considering the influence of error factors, the radius R of the sphere of the collision model is the spacing safety margin, as shown in Figure 5.

As shown in Figure 5, we have determined a range of spacing safety margins for the airplane. Error generation is random and uncertain, so it is necessary to use the error as a range to determine the safety margin ε. Therefore, we introduced statistical methods to determine the distribution characteristics of the errors under different flight conditions by analyzing a large amount of flight data. Specifically, we used Monte Carlo simulation to calculate the probability density function of the error distribution. This not only improves the accuracy of the error assessment but also provides a solid data basis for the determination of the safety margin. We considered the effects of multiple environmental factors on the error, including flight speed, flight altitude, and the accuracy of the navigation system. By synthesizing these factors, we established a more complex error safety margin model that can predict the error margin more accurately under different scenarios. The specific formulas are as follows:

(7) $$L=2R=2(R_{\mathrm{aircraft}}+R_{\mathrm{safety}\;\mathrm{margins}})$$

(8) $$\varepsilon =Z\sqrt{\sigma_{1}^{2}+\sigma_{2}^{2}+\sigma_{3}^{2}+\ldots }$$

where L is the safe distance between the two machines and $\sigma_{n}$ represents the error of various factors.

**Figure 6:**
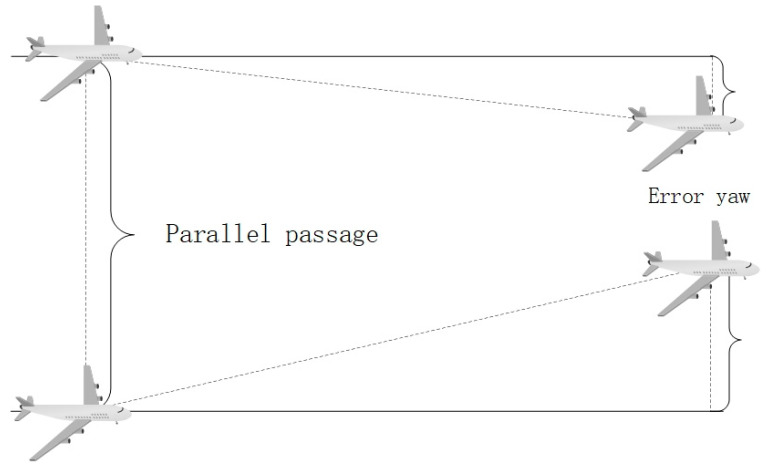
Positioning deviation schematic.

At this time, assume that the initial altitude of the two aircraft is $d_{0}$, and in time Δt, the error of the manned aircraft by the navigation performance is $\varepsilon_{1}(\sigma_{n})$ and can be denoted as $d_{0}∼d_{1}$$\left(0,\sigma_{1}^{2}\right)$, the positioning error of the UAV due to the GPS performance environment is $\varepsilon_{2}$ and can be denoted as $d_{1}∼d_{2}(0,\sigma_{2}^{2})$, and the positioning error of the UAV and the manned aircraft due to the wind speed is $\varepsilon_{3}$ and can be denoted as $d_{2}∼d_{3}(0,\sigma_{3}^{2})$. As shown in Figure 6, the actual lateral distance of the UAV is:

In this paper, it is assumed that the initial positions of the two aircrafts are at A and B, respectively, and the lateral distance between the two aircrafts is denoted as $d_{0}$; the lateral distance between the two aircrafts is denoted as $d_{1}$ due to the presence of positioning errors in the manned aircraft navigation performance (RNP), the lateral distance between the two aircrafts is denoted as $d_{2}$ after the addition of the unmanned aircrafts due to the factor of the GPS positioning errors, and the final lateral distance between the two aircrafts is denoted as $D_{3}$ due to the influence of the wind factor. It should be noted that the figure shows only the theoretical analysis, i.e., all the positioning errors lead to the shortening of the distance between the two aircrafts, thus increasing the risk of collision.

At this point, the true distance between the two aircraft can be calculated from Equation (9) as:

(9) $$D_{3}=d_{0}-\varepsilon_{1}-\varepsilon_{2}-\varepsilon_{3}$$

(10) $$d_{1}=d_{0}-\varepsilon_{1}$$

(11) $$\varepsilon_{R}=\varepsilon_{1}+\varepsilon_{2}$$

where $\varepsilon_{R}$ is the total error, and $\varepsilon_{1}$, $\varepsilon_{2}$ are the errors of the UAV due to GPS and wind speed, respectively.

Assuming that $d_{0}$ is the initial distance between the two aircraft, then $D_{3}$ satisfies:

(12) $$D_{3}∼d_{0}+N(0,\sigma_{1}^{2}+\sigma_{2}^{2}+\sigma_{3}^{2})$$

(13) $$D_{3}∼N(d_{0},\sigma_{1}^{2}+\sigma_{2}^{2}+\sigma_{3}^{2})$$

At this point, the probability density distribution function of the actual distance $D_{3}$ of the aircraft can be expressed as:

(14) $${f(D}_{3})=\frac{1}{\sqrt{2\pi (\sigma_{1}^{2}+\sigma_{2}^{2}+\sigma_{3}^{2})}}e^{\left[-\frac{{\left(D_{3}-d_{0}\right)}^{2}}{3(\sigma_{1}^{2}+\sigma_{2}^{2}+\sigma_{3}^{2})}\right]}$$

Based on the above analysis, substituting the model, the collision risk model of UAV and manned aircraft after incorporating the error is:

(15) $$N_{y}=\int_{L_{2}}^{L_{1}}f(S_{y})dD_{y}\cdot 2E_{x}P_{x}\frac{\lambda_{z}}{S_{z}}\frac{v_{y}}{2\lambda_{y}}(1+\frac{2v_{z}\lambda_{y}}{2v_{y}\lambda_{z}})(1+\frac{2v_{x}\lambda_{y}}{2v_{y}\lambda_{x}})$$

where $P_{x}$ denotes the probability that the collision box of the above equation traverses the spacer layer laterally.

## 3. Error Analysis

### 3.1. Manned Aircraft Navigation Error Analysis

Positioning errors in manned aircraft flights are mainly affected by CNS performance (communication, navigation, and surveillance performance); therefore, it is crucial to analyze the positioning errors caused by CNS [34]. Among them, CNS performance includes: required communication performance (RCP), required navigation performance (RNP), and required surveillance performance (RSP). In our team’s preliminary research on Zigong Navigation Airport, it was found that due to the weak communication and surveillance equipment of Zigong Navigation Airport compared to transportation airports, and for the small impact of aircraft-to-flight positioning error, this study focuses on analyzing the impact of navigation performance (RNP) on positioning error.

**Figure 7:**
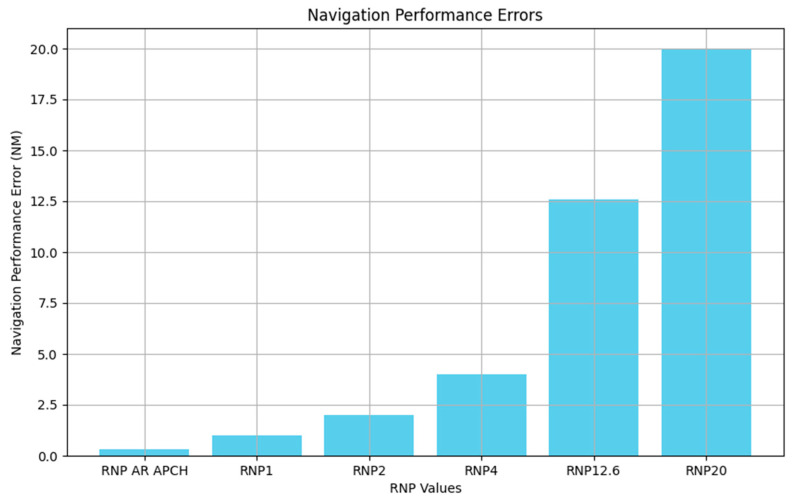
RNP accuracy comparison chart.

Navigation System Error (NSE) is known as Position Estimation Error (PEE), where RNP is a type of required navigation performance (RNP) for an aircraft flying on a route based on a combination of factors such as airports, aircraft, and other considerations [35]. The required navigation performance is mainly utilized to classify the airspace type using navigation performance accuracy, and several types of RNP are listed in Table 1. They are analyzed as follows. See Figure 7.

According to the definition of RNP in Table 1, it is known that the lateral total systematic error must be within ±a nautical mile for at least 95% of the total flight time during operations in the airspace or on the flight path designated as base RNP 1 [36]. Therefore, according to this definition, it follows that:

(16) $$\int_{-av}^{av}\frac{1}{\sqrt{2\pi \sigma_{a}}}e^{-\frac{x^{2}}{2\sigma_{a}^{2}}}dx=0.95$$

where a is the value of the parameter of RNP, i.e., navigation accuracy, which represents the localization error caused by the navigation performance.

**Figure 8:**
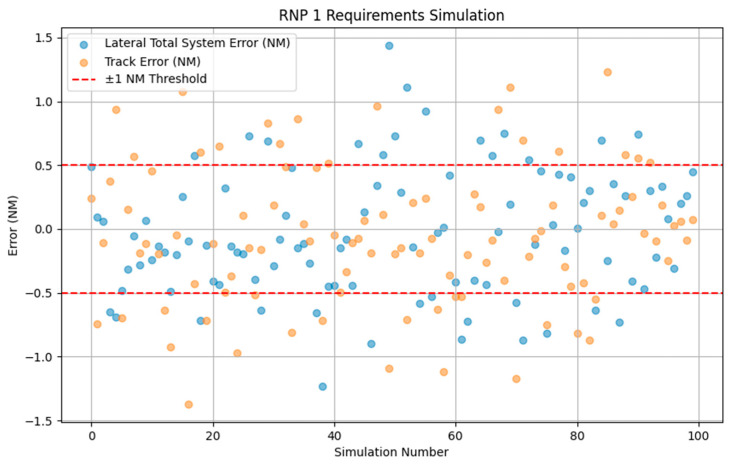
RNP1 error simulation diagram.

At this point, taking RNP1 as an example, the navigation performance error satisfies the normal distribution law, and we can visualize the error as shown in Figure 8.

When the value of RNP (required navigation performance) is taken as 1, the simulation of the positioning error is shown in Figure 8. The figure shows the changes in the Lateral Total System Error (Lateral TSE) and Track Error (Track Error) of the navigation system for 100 simulations with RNP1. Each point in the graph represents one simulation result, the horizontal axis is the number of simulations, and the vertical axis is the error value (in nautical miles). The red dashed line represents the ±1 nautical mile threshold as a tolerance for navigation performance. All error points that are between the red lines indicate that the system meets the ±1 nautical mile navigation performance requirement. If there are error points outside the red line, it means that some simulation results do not meet the requirements. Through this chart, the error distribution of the navigation system under different simulation times can be visualized. Over time, the positioning error of the aircraft does not show an obvious cumulative effect, but it shows strong instability and randomness. This characteristic stems from the fact that the RNP adopts advanced navigation equipment and technology that can monitor and correct the position of the aircraft in real time, thus effectively avoiding the accumulation of errors.

### 3.2. UAV Navigation Error Analysis

Currently, UAVs mainly rely on GPS (Global Positioning System) for navigation, while the utilization of BeiDou satellite navigation is also gradually increasing [37]. The errors of the GPS navigation system mainly originate from three aspects: the space part, the control part, and the user part. The impact of these error sources on navigation accuracy is analyzed, and they are uniformly attributed to the pseudo-range of each satellite, i.e., the user equivalent distance error (UERE) [38]. Typically, the error components of GPS are independent of each other, as shown in Table 2.

Considering the normal situation, this paper takes the GPS navigation system equipped with SA radar as an example. According to the principle of GPS positioning error, it can be seen that the UAV GPS positioning error $\varepsilon_{gps}$ obeys the Gaussian random distribution law of variance $\sigma_{gps}$, i.e., the variance is $\sigma_{gps}=\sqrt{4\times 33^{2}}=66.6$ m, and its density function class is expressed as:

(17) $$f(\varepsilon )=\frac{1}{\sqrt{2\pi }\sigma }\exp\left(-\frac{\varepsilon^{2}}{2\sigma^{2}}\right)$$

**Figure 9:**
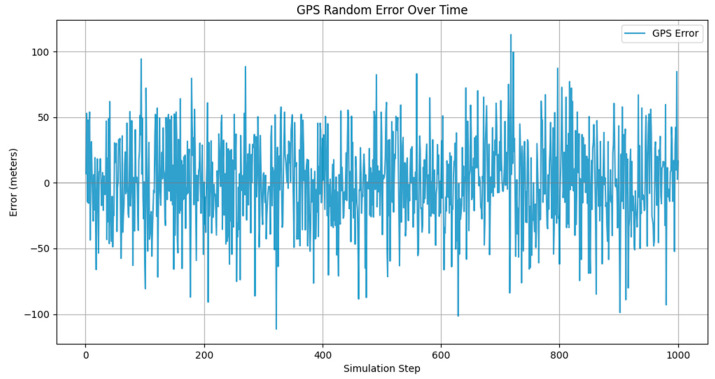
GPS error simulation chart.

The positioning errors caused by the UAV GPS can also be visualized by the tool as shown in Figure 9.

As can be seen in Figure 9, the distribution of error data points in the graph is around the 0 − axis line, which is consistent with the characteristics of the random error distribution. Most of the error values are concentrated within ±33.3 m, which is consistent with a normal distribution with a standard deviation of 33.3 m. The absolute value of the error values fluctuates around a maximum of 66.6 m but very rarely approaches this limit. This suggests that although the maximum error is set at 66.6 m, in the actual simulation, a very large majority of the errors are concentrated in a much smaller range. Furthermore, the error fluctuates randomly over time (number of simulations) with no apparent trend or periodicity. This suggests that the errors are independent for each simulation, consistent with the characteristics of random errors.

### 3.3. Wind Speed Error Analysis

**Figure 10:**
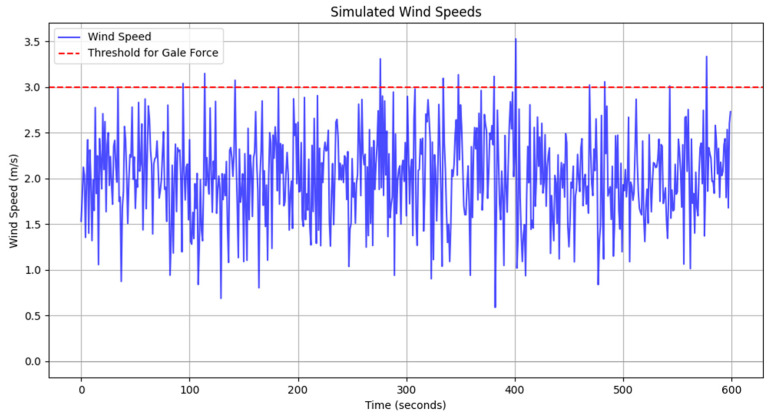
Wind speed error simulation chart.

Wind is an important consideration for flight when considering the impact of meteorological conditions on operations at navigable airports. The takeoff and landing of manned aircraft and the flight path phase can be disturbed by wind speed, which can be serious and even cause accidents; compared with manned aircraft, UAVs are more susceptible to wind speed and localization offset due to the field and their own equipment [39]. Assuming that the initial position of manned aircraft A is $D_{1}$, and the speed is $V_{1}$, and the initial position of unmanned aircraft B is $D_{2}$, and the speed is $V_{2}$, a stochastic differential equation can be used to represent the flight position state of the two airplanes in any time ‘t’:

(18) $$D_{1}=V_{1}(t)+dW_{1}(t)$$

where $D_{1}$(t) denotes the position state of a manned aircraft at moment ‘t’ in the airspace; $W_{1}$(t) is the basic Wiener process. Correspondingly, the position state of airplane 2 at any moment t can also be expressed as: $D_{2}=V_{2}(t)+dW_{2}(t)$. Considering the randomness of wind speed, this paper takes the test area airport as an example to consider the positioning error caused by the actual wind speed. The observation data of the Zigong airport ground automatic observation system are used, and the system analyzes and summarizes the change characteristics of the ground wind field with the 10 min Pinjun wind speed data at the whole point, as shown in Figure 10.

Figure 10 shows the stochastic nature of wind speeds, which vary irregularly over time. The wind speed fluctuates between 1.5 and 2.5 m/s, and in a few cases, the wind speed approaches or exceeds the 3 m/s threshold. This randomness directly affects the positioning accuracy of the aircraft, but the positioning errors of the aircraft show a relatively stable trend, consistent with the actual situation. The wind speed data have no obvious trend or periodicity and exhibit random fluctuations, consistent with the randomness of actual changes in wind speed. Due to the uncertainty of wind speed, the flight performance and safety of the aircraft are often challenged, so further research is needed to investigate the mechanism of the influence of meteorological conditions on the flight process and to seek effective coping strategies.

## 4. Computing and Simulation

### 4.1. Example Calculations

**Figure 11:**
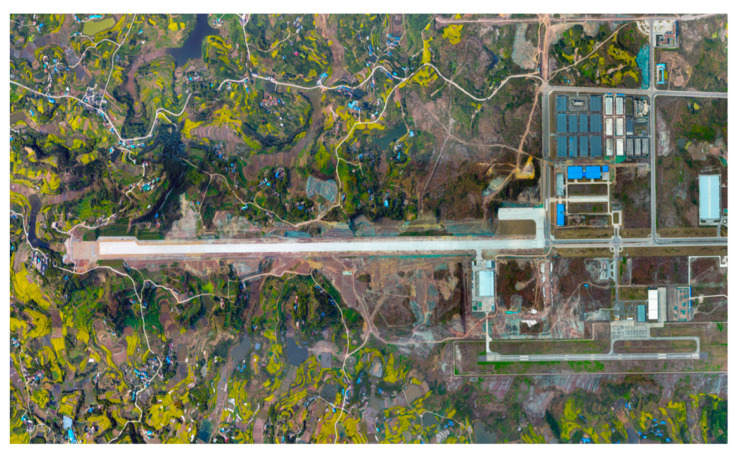
Schematic diagram of the airport.

Experimental condition assumptions:

(1) This study is only a theoretical analysis and does not change the present operating regulations of the airport;
(2) It is assumed that the error factors affecting the aircraft are independent of each other and do not interfere with each other;
(3) The research scene of this paper is the fusion operation test area of Feng Ming Airport in Zigong, as shown in Figure 11.

Taking Zigong Feng Ming Airport as the test area, it can be seen from Figure 11 that two runways with 550 m spacing are set up at this airport, which fully meets the runway conditions for the fusion operation of manned aircraft and unmanned aircraft. Moreover, Zigong Feng Ming Airport undertakes the task of the fusion operation test area, and the airspace equipped with Chuanxie 5 airspace fully meets the airspace condition requirements of the fusion operation test.

**Figure 12:**
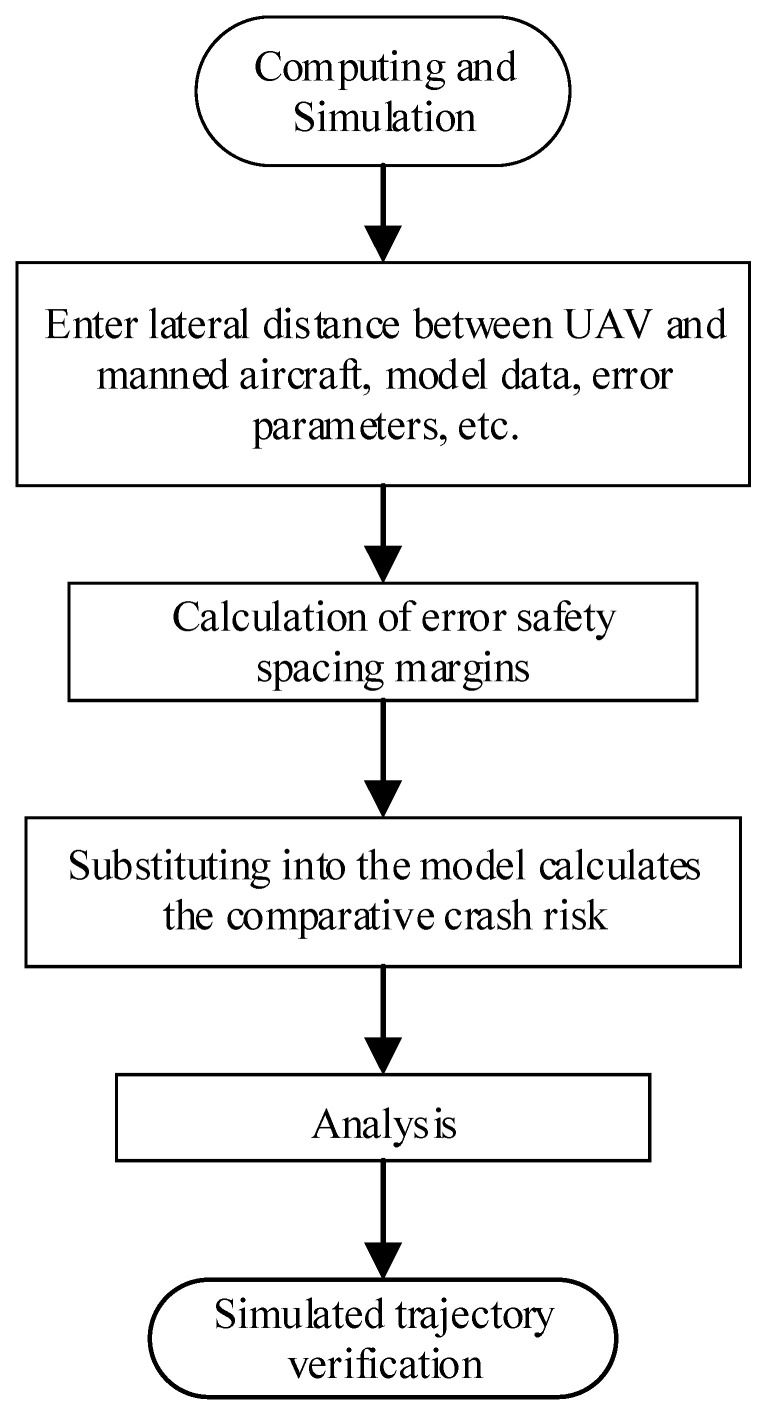
Computational simulation flowchart.

This study takes the lateral spacing of two aircraft during the fusion operation in the flight path phase as an example. The specific steps are as follows: based on building a collision risk model based on error safety spacing margin in python, substitute data (including lateral spacing, size, cruise speed, navigation error, wind speed error, lateral direction overlap rate, etc.), calculate the collision risk under the influence of different intervals of the maximum error factors, and analyze the results of the simulation to verify whether the trajectory is in line with the actual result. The specific solution process is shown in Figure 12 below.

The Zigong Feng Ming Airport statistics of manned and unmanned aircraft operation data are placed in the same field, which has a significant meaning for the collision risk assessment; we selected the 2023 UAV and manned aircraft flights statistics as shown in Table 3, flights and schedules.

Zigong Feng Ming Navigation Airport was selected as the real operation scenario, and the UAV flight data in the field in 2023, which has a total of five airspaces, in which the total number of large UAVs is 40, the total flight mission of large UAVs is about 1200 h per year, the time of the activities in the airspace is three-fifth of the total time, and the frequency of the UAVs’ flights is:

(19) $$\beta =\frac{1200\times 40}{365\times 24}\times \frac{3}{5}\times \frac{1}{5}=0.657$$

The manned training aircraft Cessna 172 and a large UAV, which have been in flight training for a long time at Feng Ming Airport in Zigong, are used as examples for the arithmetic analysis, and the name of the UAV model is not disclosed in this paper because of confidentiality. The UAV is equipped with two three-bladed engines, leading the nation in terms of power, and has a range of about 6000 km. The manned aircraft and the UAV take the flight program RNP-1 to fly, and the lateral navigation and positioning error is 1.852 km at most. Python is applied to construct and calculate the model, and the values of the model parameters are shown in Table 4.

**Figure 13:**
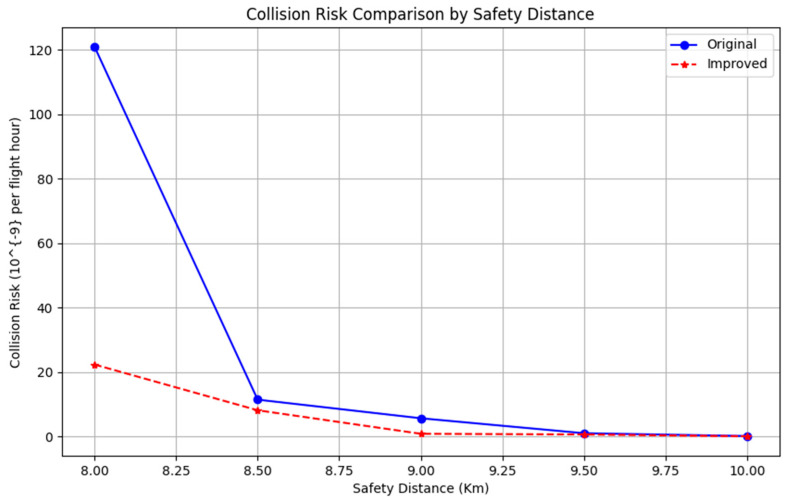
Results’ comparison chart.

When the UAV and manned aircraft are fused and operated with reference to the facilities of Feng Ming Airport in Zigong, the communication, navigation, and surveillance performance (CNS performance) are: RCP10, RNP1, and RSP1, respectively. Therefore, based on the selected manned and unmanned aircraft data, the results of the calculations are as shown in Table 5, and the results are visualized and compared in Figure 13.

**Figure 14:**
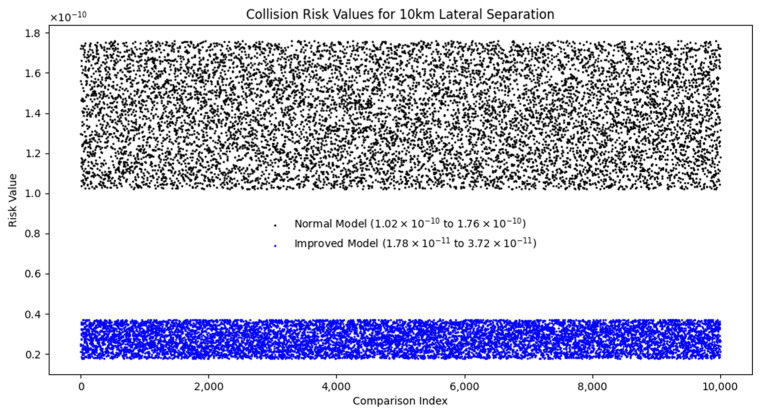
Comparison chart for 10 km calculation.

Taking the lateral spacing of 10 km as an example, we substitute the error spacing margin into Event and the improved Event model for calculation and comparison. Because the error spacing margin has a certain randomness, we carry out the simulation 10,000 times with the error safety spacing margin simulation calculation, and take the maximum collision risk value, so that the theoretical calculation results have experimental validity. Figure 14 shows the simulation results.

Figure 14 illustrates the range controllability and stability exhibited by a model that incorporates an error-safe spacing margin in the computational process. Specifically, the error-safe spacing margin enhances the performance of the model by effectively fitting and unifying the random errors. Compared with the traditional model, the improved model computes smaller errors and more focused results, while the results of the traditional model are more dispersed. This not only indicates that the improved model has a significant advantage in terms of validity but also shows that its calculation results are more accurate. The same results also appear for calculations at different lateral spacings, and the advantages of the model are demonstrated here using only 10 km, without going into too much detail. The improved model has higher stability and accuracy in handling errors, which further validates its advantages in practical applications.

ICAO, the International Civil Aviation Authority, stipulates that the target safety level in lateral, vertical, and sideways directions should not be less than $TLS=1.0\times 10^{-7}$, and the total target safety level should not be less than $TLS=5.0\times 10^{-9}$. As shown in Table 5 and the results in Figure 12, the results sought are in accordance with the target safety level. Comparing the calculation results of the Event model and the improved model, the improved model reduces the redundancy of the calculation, the probability of collision risk is lower, and it is more suitable for the model selection of the fusion operation of manned and unmanned aircraft.

### 4.2. Simulation Analysis

**Figure 15:**
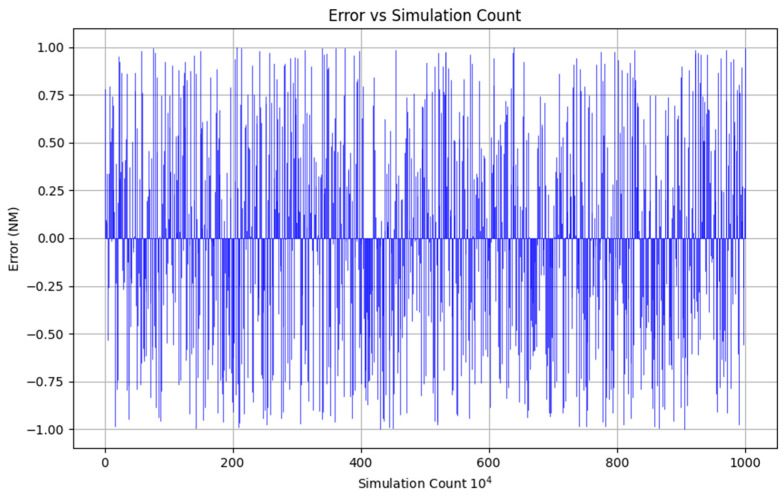
Error simulation diagram.

Considering the randomness of the error, there will be a certain error in the results of each of our calculations, and the calculation results need to be repeatedly verified several times. Therefore, considering the randomness of the error data as well as the fitting, we use the Monte Carlo method of simulation to carry out times of error simulation, as shown in Figure 15.

Figure 15 illustrates the magnitude of the random error for each of the 1000 simulations. The errors are uniformly distributed in the range of −1 to 1. Each blue line in the figure indicates the magnitude of the error for one simulation. Most of the error values fluctuate within a small range with no obvious concentration trend, which is due to the random nature of the errors themselves, consistent with the previous study. As the number of simulations increases, the error values vary randomly with no obvious trend or periodicity. This is consistent with the characteristics of random errors, where each simulation is independent and there is no tendency for the errors to accumulate or decrease over time.

In order to make the experimental effect clearer and more obvious, we compare the simulation scenarios in two extreme cases, simulating a section of the flight path running at a headroom below 3000 m, where the UAV and manned aircraft are fused to operate, with the conditions as shown in Table 6.

**Figure 16:**
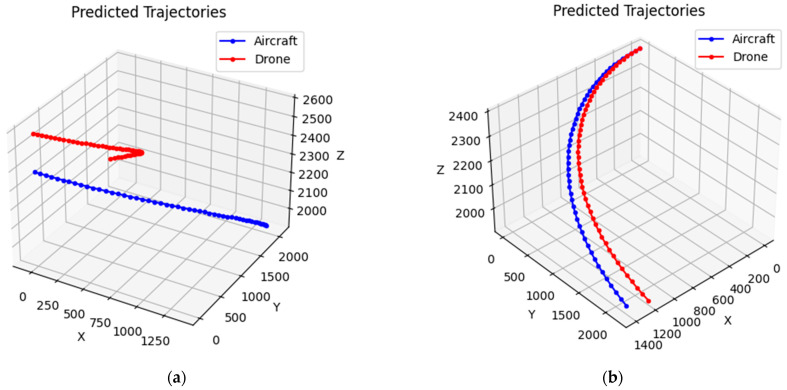
(**a**) Error-free trajectory charts; (**b**) various error trajectory charts.

The fusion operation in Table 6 maintains a safety spacing of 10 km, and under normal navigation, we simulate the prediction based on the actual operation trajectory of Zigong Airport. The numbers in Figure 16 do not represent specific meanings and units, and are only for the convenience of seeing the trend of the aircraft trajectory; the red trajectory indicates the UAV, and the blue indicates the manned aircraft. From Figure 16a, it can be seen that when there is no influence of error factors, the fusion of unmanned aircraft and manned aircraft always maintains the normal lateral safety spacing, and there is no dangerous situation in the future trajectory simulation prediction, which is consistent with the theoretical calculation results.

When the fusion operation is shortened to a safe spacing of 8 km in Table 6, and the navigation error of manned aircraft and UAVs, and the meteorological condition error are added, we make a trajectory simulation prediction based on the actual operational trajectory of the airport. From Figure 16b, we can see that when shortening the safety spacing of aircraft and adding the error factor, the fusion operation of manned aircraft and unmanned aircraft at a certain period of time in the future cannot maintain the normal lateral safety spacing due to the existence of the error factor and there is a dangerous collision situation in the future trajectory simulation prediction, which is in line with the theoretical calculation results.

## 5. Conclusions

The results show that when the number of collisions is equal to the target safety level, the corresponding two-aircraft spacing is the minimum lateral safety spacing. The higher the localization accuracy, the smaller the minimum safety spacing. From the experimental results above, it is shown that with the improvement in positioning accuracy, the obtained safety spacing may be reduced accordingly, and 8 km meets the spacing standard of the fusion running operation of manned and unmanned aircraft under suitable positioning conditions. This also proves the feasibility of the safety spacing of 10 km between manned and unmanned aircraft, which is the lateral spacing stipulated by Zigong Feng Ming Airport.

In terms of the fusion operation of manned and unmanned aircraft, we believe that it can follow the combination of theoretical and practical operation methods. After analyzing the research on collision risk, a model more applicable to the fusion operation of manned and unmanned aircraft has been proposed from the perspective of computational rationality and redundancy. Unlike the traditional collision risk model, this method designs a more rational model based on the aircraft. Starting from the fusion operation of manned aircraft and UAVs, we incorporate the influence of navigation, GPS, and wind speed positioning errors under the existing collision risk model to form an improved collision model for the fusion operation, which is combined with the actual operation data for value calculation. The final simulation results show that this theoretical research method has some reference value for the new scenario of manned aircraft and UAV fusion operation. However, there are still some issues that need to be improved and further researched, including practical research and algorithm optimization.

## 6. Outlook

At present, the research on the fusion operation of manned and unmanned aircraft at Feng Ming Airport in Zigong is in the experimental stage, and due to a variety of factors such as time, the complexity of the experimental environment, the precision of the equipment, the size of the research team, and the individual skills, there are some limitations in the data of the paper in terms of the error, the model, and the model algorithm. In view of this, we propose the following points as the direction for future in-depth research.

(1) Model: Future research can further expand the scope of application of models and consider more types of fusion operation scenarios of manned and unmanned aircraft. In particular, as technology evolves and new UAV designs emerge, research could focus on the performance of these new models in fusion operations and their synergistic performance with traditional manned aircraft. In addition, the fusion of vehicles of different scales, speeds and missions could be considered to explore a wider range of fusion operation possibilities.
(2) Model algorithms: Future research could further optimize the algorithms in the collision risk model to improve the accuracy and reliability of the model. For example, more complex navigation and wind speed positioning error models can be introduced to take into account the influence of more environmental factors to more realistically reflect the risk situation in fusion operation. At the same time, new data fusion techniques and machine learning methods can be explored to improve the generalization ability and applicability of the model by using more actual operational data for model training and validation.
(3) Application scenarios: In addition to theoretical research and model algorithm optimization, future research can also focus on the application and validation of fusion operation in practical application scenarios. For example, in the fields of urban air traffic management, disaster rescue, and military combat, the advantages and challenges of the fusion operation of manned aircraft and UAVs can be explored, and corresponding solutions and policy recommendations can be put forward. At the same time, we can cooperate with partners in related fields to build experimental platforms and test bases to verify the effectiveness of new theories and algorithms, and to promote the practical application and popularization of fusion operation technology.

## Figures and Tables

**Table 1:** RNP performance parameters.

Typology	Accurate (NM)	Scope of Application
RNP AR APCH	≤0.3 NM	Allow for flexible routing
RNP1	±1 NM	Terminal area approach and departure RNP APCH start, intermediate, and re-flight phases
RNP2	±2 NM	Domestic land routes
RNP4	±4 NM	Oceans and remote continents
RNP12.6	±12.6 NM	Maritime airspace
RNP20	±20 NM	ATS providing minimum airspace traffic

**Table 2:** Table of satellite navigation errors.

Affiliated Units	Error Source	System Navigation Error (With SA)	System Navigation Error (Without SA)
Space segment	Satellite clock stability	3.0	3.0
	Satellite perturbation determinability	1.0	1.0
	Select available (SA)	32.3	0
	Else	0.5	0.5
Control section	Anticipated errors in the ephemeris	4.2	4.2
	Other (Thrust performance)	0.9	0.9
	Ionospheric delay	5.0	5.0
User section	Tropospheric delay	1.5	1.5
	Receiver noise	1.5	1.5
	Multipath error	2.5	2.5
	Else	0.5	0.5
System UERE	Total error	33.3	8.0

**Table 3:** Flight movements and schedules.

Table of Statistics on Sorties for the Year 023				
Date	Total Number of Sorties	Total Time	Drone Sorties	Drone Time
2023	46,094	23,127	638	1480

**Table 4:** Model parameter values.

Data	Secrecy Drone	Cessna 172
Cruising speed V	220 km/h	229 km/h
Presses $\lambda_{y}$	3.3 m	2.72
Wingspan $\lambda_{x}$	20 m	11 m
Fuselage $\lambda_{z}$	10 m	8.28 m
Temp T	10 °C	10 °C
Maximum flight altitude H	8000 m	4267 m
Vertical proximity ratio $E_{x}$	0.61	0.61
Vertical overlap probability $P_{z}$	0.5	0.5
Lateral interval loss rate $G_{y}$	$4.8\times 10^{-6}$	$4.8\times 10^{-6}$
Navigational error $\sigma_{n}/\sigma_{gps}$	66.6 m	≤1.852 km
Wind speed error $\sigma_{w}$	≤3 m/s	≤3 m/s

**Table 5:** Calculation result.

Data	Event Model	Improvements to the Event Model
10 km	$1.02\times 10^{-10}$	$1.78\times 10^{-11}$
9.5 km	$9.71\times 10^{-10}$	$5.89\times 10^{-10}$
9 km	$5.62\times 10^{-9}$	$7.98\times 10^{-10}$
8.5 km	$1.14\times 10^{-8}$	$8.10\times 10^{-9}$
8 km	$1.21\times 10^{-7}$	$2.23\times 10^{-8}$

**Table 6:** Simulation condition.

Safety Distance	Error Condition
10 km	Error-free
8 km	Various factors error

## Data Availability

The data that support the findings of this research are available from the author, C.H., upon reasonable request.
